# The Behavior of *Listeria monocytogenes* During the Shelf Life of Wiener Sausages, as an Effect of Fermented Parsley Root Juice and Hawthorn Berry Phenolics

**DOI:** 10.3390/foods14091513

**Published:** 2025-04-26

**Authors:** Georgeta Ștefan, Gheorghe Valentin Goran, Corina Nicoleta Predescu, Maria Rodica Gurău, Stelian Bărăităreanu

**Affiliations:** 1 Clinical Science 1 Department, Faculty of Veterinary Medicine of Bucharest, University of Agronomic Sciences and Veterinary Medicine of Bucharest, 105 Splaiul Independentei, District 5, 050097 Bucharest, Romania; georgeta.stefan@fmvb.usamv.ro (G.Ș.); maria.gurau@fmvb.usamv.ro (M.R.G.); stelian.baraitareanu@fmvb.usamv.ro (S.B.); 2 Paraclinical Science Department, Faculty of Veterinary Medicine of Bucharest, University of Agronomic Sciences and Veterinary Medicine of Bucharest, 105 Splaiul Independentei, District 5, 050097 Bucharest, Romania; 3 Preclinical Science Department, Faculty of Veterinary Medicine of Bucharest, University of Agronomic Sciences and Veterinary Medicine of Bucharest, 105 Splaiul Independentei, District 5, 050097 Bucharest, Romania; corina.predescu@fmvb.usamv.ro

**Keywords:** *Listeria monocytogenes*, fermented parsley root juice, hawthorn berry phenolics

## Abstract

The behavior of *Listeria monocytogenes* (*L. monocytogenes*) throughout the shelf life of ready-to-eat foodstuffs represents a major concern in relation to human diet and human health. The aim of the study was to evaluate the behavior of *L. monocytogenes* in Wiener sausage, as an RTE meat product, throughout 15 days of storage (0–7 °C) under the action of fermented juice from parsley (*Petroselinum crispum* var. *tuberosum*) roots and common hawthorn (*Crataegus monogyna*) berry phenolics, compared with the effect of the food additives sodium nitrite and sodium ascorbate used in the standard formulation. For this purpose, one experimental formulation (F1) and one standard formulation (F2) of Wiener sausages were designed using the following preservatives and antioxidants: 50 ppm fermented parsley root juice (as a nitrite source) and 50 ppm hawthorn berry phenolics were used in F1, and 50 ppm sodium nitrite (as food additive E 250) and 50 ppm sodium ascorbate (as food additive E 301) were used in F2. The ability to support *L. monocytogenes* growth was assessed by a challenge test throughout the 15 days of storage. Based on the results of the assessment, the natural ingredients fermented parsley root juice and hawthorn berry phenolics could act as preservatives that ensure microbiological safety during the shelf life of the product. The nitrite and phenolic compounds of these natural ingredients showed antimicrobial activity against foodborne pathogens, including *L. monocytogenes*.

## 1. Introduction

*Listeria monocytogenes* (*L. monocytogenes*), one of the most important foodborne pathogens causing listeriosis in risk groups of the human population—especially newborns, pregnant women, immunosuppressed people, and the elderly—represents a major concern regarding public health. It is accepted that human listeriosis is mainly caused in susceptible people by the ingestion of contaminated food, with the minimum infectious dose being unknown [1,2]. The clinical presentation depends on the infected dose, the characteristics of *L. monocytogenes*, and the immunological status of the infected person [3]. It causes serious pathologies in humans, including meningitis, septicemia, encephalitis, and abortion [3,4]. In the past twenty years, *L. monocytogenes* has been a major problem for the food industry, particularly ready-to-eat (RTE) foods, due to its ubiquitous nature, its ability to survive in adverse conditions (e.g., refrigerated conditions), and the possibilities to contaminate RTE meat products during the manufacturing process [5]. The contamination can be caused by various sources during the manufacturing process of RTE meat products, which can be a real problem in conditions of low-level hygiene [1]. The potential of *L. monocytogenes* contamination post-heat treatment through the production environment is a significant risk, particularly during the cooling and packaging stages. It can survive and grow during the shelf life of the products under appropriate conditions [6]. It can grow at the storage temperatures of meat products (between 0 °C and 7 °C), in conditions of water activity (a_w_) ≥ 0.94 of the substrate, and in the pH range between 5 and 9.4. Due to the ability to grow in suitable conditions, *L. monocytogenes* is considered a potential risk for public health as a foodborne pathogen. The maximum level of *L. monocytogenes* in foodstuffs should be below 100 CFU/g product [7]. A low level may need to be achieved at the manufacturing stage of foodstuffs to ensure that this level is not exceeded at the time of consumption, as bacterial growth may occur during shelf life.

The microbiological food safety criteria are laid down in European Regulation (EC) No. 2073/2005 for certain microorganisms in foodstuffs. Regarding *L. monocytogenes* in RTE foods, except those intended for infants and special medical purposes, two criteria are available, based on the ability of food to support *L. monocytogenes* growth. For RTE foods capable of sustained growth, it is mandatory to assess the presence/absence of *L. monocytogenes* before the food has left the immediate control of the food business operator by detection, and it is considered compliant if *L. monocytogenes* is absent/25 g product. For those unable to sustain growth, a count of *L. monocytogenes*/g product is mandatory and must not exceed 100 CFU/g throughout the shelf life of the product [8].

The risk assessment regarding the ability of RTE foods to support *L. monocytogenes* growth is mandatory, according to legal provisions. For this purpose, RTE food business operators shall perform studies to assess the growth of *L. monocytogenes* that could be present in food over its shelf life and to ensure compliance with microbiological criteria [8]. One of these is the challenge test, a microbiological study to assess the growth of *L. monocytogenes* in artificially contaminated food maintained in predictable conditions.

Wiener sausages, one of the most popular products, are heat-treated meat products, requiring no heat treatment before consumption. Its physicochemical properties, i.e., pH and water activity (a_w_), as well as the storage temperature (0–7 °C), provide suitable conditions for the viability and multiplication of *L. monocytogenes* during the shelf life of the product. To guarantee microbiological stability through shelf life and to prevent the risk of transmission of *L. monocytogenes* through their consumption is a constant challenge for the meat industry. Various synthetic compounds are used in the meat industry to maintain the organoleptic properties and to reduce or inhibit the growth of spoilage and pathogenic bacteria, either as preservatives (e.g., potassium/sodium nitrite) or as antioxidants (e.g., sodium ascorbate). Meanwhile, there is a growing consumer interest in products that contain mostly or exclusively natural ingredients. The possible side effects of food additives on human health are also an issue of concern, such as asthma, allergies, etc. [9,10]. For these reasons, operators in the meat industry are concerned with the identification of natural ingredients, as preservatives that can provide organoleptic, physicochemical, and microbiological stability of meat products during their shelf life [11]. The natural alternatives, such as different herbs, vegetables, and fruits, are the traditional practices that can also be used on an industrial scale.

A range of vegetables, fruits, and herbs are suitable alternatives to sodium ascorbate in meat product technology due to their phenolic content. The antioxidative and antimicrobial effects as a result of the use of vegetable and fruit extracts rich in phenolic compounds have been demonstrated in many studies [12,13,14]. The antimicrobial action of polyphenols is based on the inactivation of various bacterial multiplication enzymes and the inhibition of biofilm formation, and has been demonstrated against foodborne bacteria [15,16]. The inhibitory effects of gallic acid on the adhesion and motility of *L. monocytogenes* were demonstrated in the studies of Borges et al. [16,17]. Gallic acid and ferulic acid cause disruption of the cell membrane and leakage of various cellular components from *L. monocytogenes*, *E. coli*, etc. [17]. P-coumaric acid and phenolic acid can disrupt cell membranes and can attach to bacterial DNA [18]. Several studies underlined the potential antimicrobial activity of hawthorn (*Crataegus monogyna*) extract against Gram-positive and Gram-negative bacteria [19,20,21]. The in vitro study of Tadic et al. indicates a mild bactericide effect of hawthorn berry extract against *L. monocytogenes* (in a disk diffusion assay) [22]. The alcoholic extract of hawthorn berries represents a valuable natural source of polyphenols with antioxidant properties that can be useful in meat preservation [23].

Replacing sodium nitrite with a variety of plant extracts is an acceptable solution, based on the results of several studies [24]. Various root vegetables such as parsley, parsnips, and celery can be a source of nitrites, which fix nitrogen in the form of nitrate. Nitrate in root vegetable juices is reduced to nitrite during fermentation in the presence of nitrate reductase-producing bacterial strains (e.g., *Staphylococcus xylosus*) [25]. *Staphylococcus* spp. have the ability to use nitrate as an oxygen source (as an electron acceptor) under anaerobic conditions when the nitrate regulatory element (Nre system) of the bacteria is activated [26]. *Staphylococcus xylosus* (*S. xylosus*), one of the coagulase-negative strains, can develop important nitrate reduction activity [27]. The study of *Papuc* et al. showed that a maximum concentration of nitrites was reached after 24 h for fermented parsley juice in the presence of strain of *S. xylosus* ATCC 29971 [28]. Among the various root vegetables studied, fermented parsley juice presented an increased nitrate content but also appropriate organoleptic properties suitable for meat products (e.g., very low levels of vegetable pigments and a mild, pleasant flavor) [28]. The antimicrobial effect of nitrite in meat products against *L. monocytogenes* is directly influenced by intrinsic and extrinsic parameters, an inhibitory action being observed under conditions of pH value < 5.5, NaCl concentration > 3%, and refrigeration storage [29]. Juntila et al. demonstrate a slight antimicrobial effect of nitrite under conditions of fermentation temperature of sausages [30].

The present study assessed the behavior of *L. monocytogenes* under conditions of replacing sodium ascorbate (as a synthetic antioxidant) with a hawthorn (*Crataegus monogyna*) berry extract (HBE), rich in phenolics, and synthetic sodium nitrite (E250) with fermented parsley (*Petroselinum crispum*) root juice (PFJ) in unsmoked Wiener sausage, based on a challenge test. These natural ingredients could be valuable alternatives in the RTE food industry due to their preservative value, including the antimicrobial effect.

## 2. Materials and Methods

### 2.1. Wiener Sausage Manufacture and Sampling

For the manufacture of Wiener sausages, two types of product formulations were used. An experimental formulation for the product was marked as F1 and the standard formulation for the product was marked as F2, with the following preservatives and antioxidants: 50 ppm fermented parsley root juice (PFJ) (as a nitrite source) and 50 ppm hawthorn berry extract (HBE) in F1, and 50 ppm sodium nitrite (food additive E 250) and 50 ppm sodium ascorbate (food additive E 301) in F2. Three batches were produced from each formulation, labeled as follows: B1, B2, and B3 for product F1; B4, B5, and B6 for product F2 (Table 1).

The ingredients of Wiener sausages included 79% of raw material, 1.6% ice flakes, 1.5% salt, 1.25% onion flakes, 0.1% garlic powder, 0.5% spices, and the formulations of preservatives and antioxidants indicated in Table 1. The preservatives and antioxidants of the standard formulation F2 were used according to the recipe of the Wiener sausage producer.

**Figure 1:**
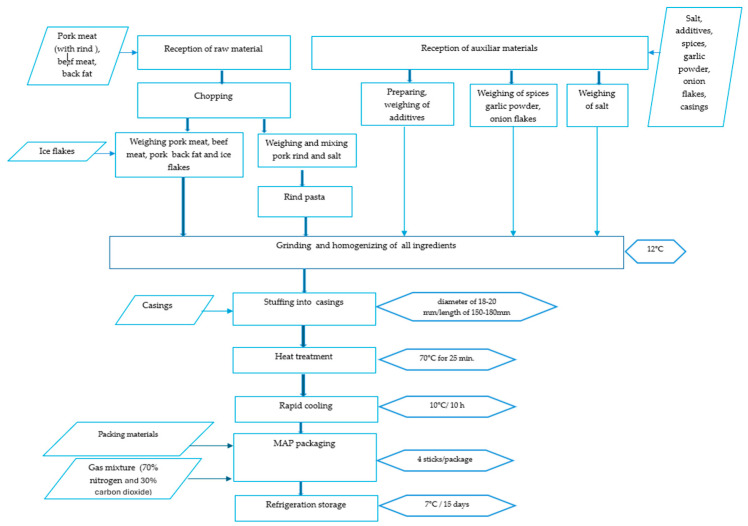
A flow chart of the Wiener sausage manufacturing process.

Wiener sausages are RTE heat-treated meat products, which contain raw meat, various spices, and food additives stuffed in casings. The manufacturing process was based on the defined technology of Wiener sausages, such as the chopping of the raw meat, weighing of ingredients, grinding and homogenizing of all ingredients into the cutting machine, stuffing of composition in casings (diameter of 18–20 mm and an average length of 150–180 mm), heat treatment stage (25 min at a minimum of 70 °C in the middle of the stick), rapid cooling (to 10 °C in the middle of the stick, in 10 h), MAP packaging, and refrigeration storage (7 °C). Prior to MAP packaging, the casings were removed. The manufacturing process described above is shown as a flow chart in Figure 1.

The raw meats used in the recipe were pork meat (with rind), and beef meat and backfat, which were purchased from local slaughterhouses. The supplier, Pacovis Romania, provided the antioxidant sodium ascorbate (food additive E301). The preservative, sodium nitrite as food additive E250, was provided by Solina Romania. The preservatives and antioxidants (PFJ, HBE, N, and A), dissolved in chilled sterilized distilled water (in a 100 mL final volume), were added during the grinding and homogenization stages into the cutting equipment, in accordance with the defined formulations (F1 and F2). Each product formulation was produced in three batches, on three consecutive production days, with each batch representing one production day (6 batches in total). The samples were packaged under modified atmosphere conditions (MAP) using a gas mixture of 70% nitrogen and 30% carbon dioxide, with each package containing 4 sticks of product.

Five time intervals were defined during 15 days of storage: T0 (day 0 of storage), T1 (3rd day of storage), T2 (5th day of storage), T3 (10th day of storage), and T4 (15th day of storage/last day of storage). This period represents the validated shelf life of Wiener sausages manufactured on standard formulation. The samples have been stored under refrigerated conditions, at 7 °C, according to the maximum level recommended by the producer for Wiener sausages for 15 days. The evaluation of *L. monocytogenes* behavior was carried out by a challenge test—the growth potential assessment according to the EURL *Lm* Technical Guidance Document on challenge tests and durability studies [31] and ISO 20976-1 [32].

### 2.2. Obtaining Fermented Parsley Juice (PFJ)

Parsley roots were purchased from a local grocery store. After washing with cold water, the roots were cut into 1 × 1 cm pieces, blended with sterile distilled water at a ratio of 1:2 (*w*/*v*), and stored at 4 °C for 3 h before being filtered and sterilized at 121 °C for 15 min. The juice fermentation was made by adding 10^8^ CFU/mL of strain of *Staphylococcus xylosus* (ATCC 29971) in an incubator at 37 °C for 36 h [33]. The suspension was passed through Whatman no. 1 filter paper. The solvent was removed using a rotary evaporator (Heidolph Laborota 4000), resulting in the fermented parsley juice (PFJ). The PFJ had a pH of 5.3 and a nitrite content of 6237.5 ppm (determined by the AOAC method) [34].

### 2.3. Obtaining Hawthorn Berry Phenolics (HBE)

The raw material, consisting of dried hawthorn berries, was sourced from the spontaneous flora and harvested in the forests of Arges County. The berries are washed and ground into a fine powder using laboratory milling equipment. The HBE was performed by maceration with ethanol 60% (*v*/*v*); the berry powder-to-solvent ratio used was 1:10 (*w*/*v*) for 5 h, followed by hot extraction in a shaking water bath (GFL 1092) at 60 °C for 3 h. The extract obtained was then passed through Whatman no. 1 paper filter. The removal of the solvent was carried out by means of a rotary evaporator (Heidolph Laborota 4000) at a temperature of less than 80 °C. Total phenolic content of HBE, expressed as mg gallic acid equivalent per mL (mg GAE/mL), was performed using the Folin–Ciocalteu methodology [35].

### 2.4. Preparation of L. monocytogenes Inoculum

A cocktail of three strains of *L. monocytogenes* was used for artificial inoculation, i.e., one strain of *L. monocytogenes* ATCC 13932 and two strains of *L. monocytogenes* isolated from meat products (*L. monocytogenes* 1 and *L. monocytogenes* 2). The inoculum preparation was performed according to the protocol of the EURL *Lm* Technical Guidance Document [31]. Two successive plating steps were carried out for each strain, the first one on Brain Heart Infusion (bioMérieux, Marcy L’Etoile, Lyon, France), incubated at 37 °C for 24 h, and the second on Tryptic Soy Broth (bioMérieux, France), incubated at 7 °C for 7 days.

The second cultures of *Lm* ATCC, *L. monocytogenes* 1, and *L. monocytogenes* 2 were mixed prior to inoculation by adding 1 mL of each strain in Eppendorf tubes, followed by serial decimal dilution in buffer peptone water to achieve the target inoculum concentration. In order to determine the inoculum level, 0.1 mL of the mixture of strains was plated on Listeria Ottaviani and Agosti agar (ALOA^®^, bioMérieux, Marcy L’Etoile, France) and incubated at 37 °C for 24 h, followed by *L. monocytogenes* counting.

### 2.5. The Artificial Inoculation of Samples

A surface inoculation of the sample was performed inside the package using a sealing septum to protect the modified atmosphere conditions [31]. Each sample unit was inoculated with a sterile graduated syringe through a septum and then shaken in order to ensure a uniform distribution of inoculum. For this purpose, a maximum of 1 mL inoculum (not exceeding 1% of sample mass) was used /sample unit. The assumed level of contamination was approximately 10^2^ CFU *L. monocytogenes*/g sample. The evaluation of the behavior of *L. monocytogenes* was carried out during 15 days of storage, at each defined time interval (T0, T1, T2, T3, and T4), by counting *L. monocytogenes*. T0 (day 0 of storage) represents the day of inoculation. In total, 7 samples were inoculated from each batch, giving a total of 42 inoculated samples, with 3 samples/batch used at each defined time point.

### 2.6. Detection of L. monocytogenes

The detection of *L. monocytogenes* was performed on three blank samples, each at T0 and T4/batch, to assess the potential contamination of the product. A total of 36 blank samples were tested. The VIDAS^®^ Listeria monocytogenes II test (LMO2 VIDAS^®^, bioMérieux, France) was used as a certified alternative method, and the equipment was the mini VIDAS^®^ (bioMérieux, France), based on the enzyme-linked fluorescence assay (ELFA) technique. The preparation sample of the LMO2 VIDAS^®^ protocol was used; for example, 25 g of samples were mixed with 225 mL of pre-enrichment broth (half Fraser broth, bioMérieux, France) in a stomacher for 50–60 s and incubated for 24 h at 30 ± 1 °C, followed by the transfer of 0.1 mL of suspension into 10 mL of enrichment broth (Fraser broth, bioMérieux, France) and subsequent incubation for 24 h at 37 ± 1 °C [36].

### 2.7. Enumeration of L. monocytogenes

The enumeration of *L. monocytogenes* was performed on the inoculated samples at five defined times, on three samples/batch at T0 and one sample/batch at T1, T2, T3, and T4. A total of 42 inoculated samples were tested for *L. monocytogenes* counting during the trial, using the standard method EN ISO 11290-22, Part 2 [37]. For the initial suspension, a 10 g sample was mixed with 90 mL buffered peptone water (bioMérieux, France) in a sterile bag, using a stomacher for 50–60 s. The initial suspension and subsequent decimal dilutions were inoculated in duplicate on selective chromogenic medium ALOA (ALOA^®^, bioMérieux, France) for 24 h at 37 °C, followed by the counting of *L. monocytogenes*. The results achieved were expressed as log_10_ CFU/g.

### 2.8. Determination of Physicochemical Parameters

The evaluation was carried out on control samples, one at T0 and one at T4, respectively, per batch. A total of 12 control samples were analyzed during the trial. The control samples were injected with a volume of sterile physiological serum (equal to the volume of inoculum) and stored under the same conditions as the inoculated samples. The water activity (a_w_) was measured using the device AquaLab 3TE (Decagon Devices, Pullman, WA, USA), based on the ISO 18787:2017 method [38].

The pH value was determined by direct measurement using a pH meter (Hanna Instruments, Cluj Napoca, Romania), with a glass electrode calibrated with the phosphate buffers 4.0 and 7.0 at 21 °C. We used 10 g of samples mixed with distilled water, in a ratio of 1:100, for 30 min, followed by filtration and pH measurement [39].

### 2.9. Enumeration of Aerobic Mesophilic Microflora (TVC)

The TVC enumeration was conducted on control samples, one at T0 and one at T4, respectively, per batch, to assess the natural contamination of products. A total of 12 control samples were analyzed during the trial. The samples were injected with a volume of sterile physiological serum (equal to the volume of inoculum and stored under the same conditions as the inoculated samples). The Tempo TVC test (Tempo^®^ AC, bioMérieux, Marcy L’Etoile, France) and the automated system Tempo^®^ (Tempo^®^, bioMérieux, Marcy L’Etoile, France) were used as a certified alternative method [40]. The results are expressed as log CFU/g.

### 2.10. Determination of Growth Potential (Δ)

Determination of growth potential was conducted for each batch, according to the EURL *Lm* Technical Guidance Document on challenge tests and durability studies for assessing the shelf life of ready-to-eat foods related to *L. monocytogenes* [31].

The growth potential (Δ) is the criterion used for RTE foods, considering 0.5 log_10_ CFU/g as the threshold value to classify according to Regulation (EC) No. 2073/2005 in two categories: category 1.2.—RTE foods capable of supporting the growth of *L. monocytogenes* when Δ is higher than the limit of 0.5 log_10_ CFU/g, and category 1.3.—RTE foods unable to support *L. monocytogenes* growth if Δ is less than or equal to the limit of 0.5 log_10_ CFU/g [31].

Δ was calculated as the difference between the highest observed *L. monocytogenes* CFU (log_10_ CFU/g) during the test and the initial *L. monocytogenes* CFU (log_10_ CFU/g) at T0 (day 0 of storage), according to the formula:

Δ = *log max* − *log I*, where *log max* is the highest value of the *L. monocytogenes* count recorded on samples at the defined time tested T1 (3rd day of storage), T2 (5th day of storage), T3 (10th day of storage), and T4 (15th day of storage/last day of storage), excluding the data at T0; and *log I* is the mean value of the *L. monocytogenes* count on the three samples at day zero (T0). The parameter growth potential *(*Δ) was calculated for each batch.

In order to classify products F1 and F2 as described above, the most critical values were used [31].

### 2.11. Statistical Analysis

Statistical measures, including mean, standard deviation, and variance, were calculated to summarize the data presented in the table. In this study, all samples from the experimental (B1, B2, B3) and standard (B4, B5, B6) groups were collected independently. Analyses were conducted using R programming language (version 4.4.3; R Core Team, 2025) on Windows 11 Pro 24H2 (build 26100), utilizing various R packages: dplyr, ggplot2, dunn.test, car, multcomp, PMCMRplus, and agricolae [41,42,43,44,45,46,47].

Before performing a one-way analysis of variance (ANOVA), several assumptions were made and preliminary analyses conducted to ensure the validity of the results. The mean, standard deviation, and variance for each set of batches were calculated using the ‘dplyr’ package to provide an overall picture of the central tendency and variability of the experimental (B1–B3) and standard (B4–B6) groups. Outliers were identified visually using the boxplot function from the ‘ggplot2’ package, as well as programmatically with the interquartile range (IQR) method.

ANOVA was employed to assess the significance of differences between the means of the distinct samples (n = 3) at a significance level of *p* < 0.05. After performing the ANOVA, we checked the normality of the residuals using the Shapiro–Wilk test and Q-Q plots, and conducted Levene’s test on the residuals to assess the equality of variances. Duncan’s multiple range test for post hoc comparisons was performed using the DuncanTest function from the ‘agricolae’ package to identify which specific groups (batches) were significantly different from one another.

In cases where Levene’s test indicated significant differences in variances across the groups, we performed a one-way ANOVA using Welch’s method, followed by the Games–Howell test for post hoc comparisons. The Kruskal–Wallis test was utilized as a non-parametric alternative to one-way ANOVA when the normality of the residuals from the ANOVA model was not met. To pinpoint specific group differences after significant results from the Kruskal–Wallis test, Dunn’s test with Bonferroni adjustments for post hoc analysis was employed. When the data did not meet the assumptions required for parametric tests, the Mann–Whitney U test was used to compare the experimental group (batches B1, B2, and B3) with the standard group (batches B4, B5, and B6). Additionally, effect sizes were considered, if necessary, to clarify the practical significance of observed differences.

## 3. Results

For each batch, the following tests occurred: aerobic mesophilic microflora count (TVC) and physicochemical parameter determination (i.e., pH and a_w_) in two control samples, *L. monocytogenes* detection in six blank samples, and *L. monocytogenes* enumeration in seven artificially inoculated samples. In total, 42 artificially inoculated samples, 36 blank samples, and 12 control samples were used in the study.

### 3.1. Control Sample Results

#### 3.1.1. Evaluation of Aerobic Mesophilic Microflora Counts

The effect of 15 days of storage under refrigeration conditions (values between 2 and 4 °C) on experimental batches (B1, B2, and B3) and standard batches (B4, B5, and B6) was evaluated through total viable count (TVC) of aerobic mesophilic microflora, expressed as log CFU/g (Table 2). TVC varied from 2.35 ± 0.40 log CFU/g to 2.50 ± 0.40 log CFU/g at T0, and from 3.23 ± 0.35 log CFU/g to 3.30 ± 0.35 log CFU/g at T4 for experimental batches B1, B2, and B3. TVC values for standard batches B4, B5, and B6 ranged from 2.37 ± 0.45 to 2.53 ± 0.35 log CFU/g at T0 and from 3.70 ± 0.30 to 3.85 ± 0.35 log CFU/g at T4. The results were expressed as mean ± standard deviation (SD).

The average total viable count (TVC) at T0 is slightly lower for the experimental group (2.43) compared to the standard group (2.47). Both groups exhibit low standard deviations, indicating consistency within each group. By T4, the mean TVC significantly increases for both groups, with the standard group showing a higher average (3.77) compared to the experimental group (3.28). Interestingly, the experimental group has a slightly lower standard deviation, suggesting less variability in counts relative to the standard group (Table 1).

**Figure 2:**
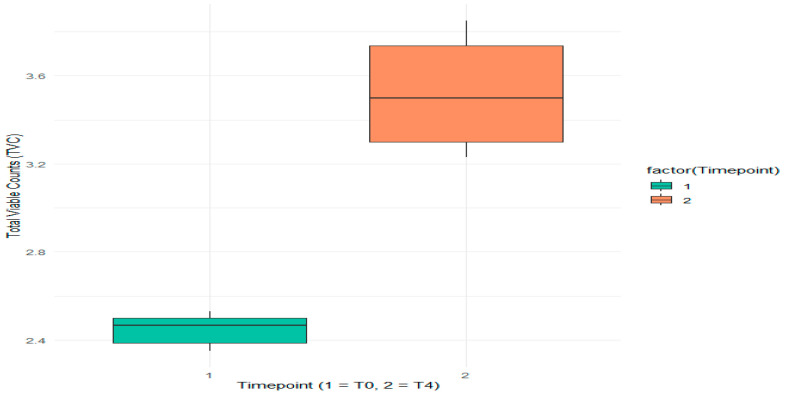
The total viable counts (TVCs) of Wiener sausages on day 0 (T0 or 1) and day 15 (T4 or 2).

A visual representation identifying outliers—defined as points outside the “whiskers” of the boxplot—was generated using the boxplot function (Figure 2) and programmatically calculated through the interquartile range (IQR) method. The boxplot effectively illustrates changes in microbial counts over time, highlighting both the increase in TVC and the variability at each time point.

The boxes show the median and interquartile range, while the whiskers indicate the range of non-outlier minimum and maximum values. There seems to be an increased average TVC at T4 compared to T0, which suggests that some microbial growth may have occurred during the storage period. Any outlier in this case would be shown as a single point beyond the whiskers.

**Figure 3:**
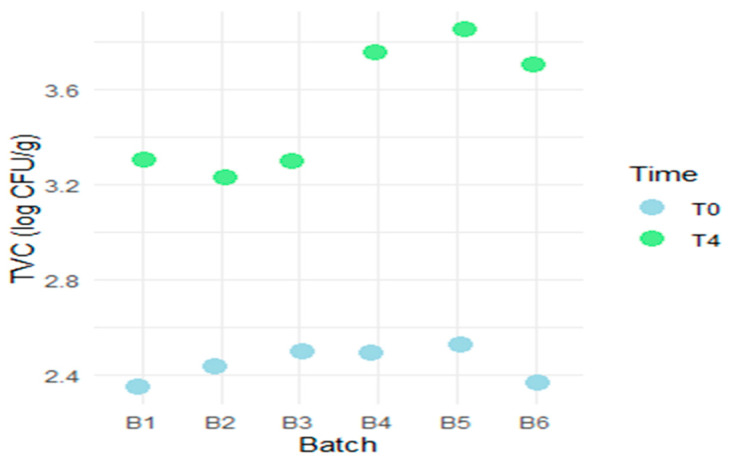
Total viable counts (TVC) measured by batch and time.

Additionally, a point plot was created to provide a clear visualization of differences across batches and time points without misrepresenting data distributions (Figure 3). This scatter plot displays the TVC measured in log CFU/g for different batches of Wiener sausages at two time points: T0 (day 0 of storage) and T4 (day 15 of storage). At T0, experimental batches (B1, B2, B3) generally start at lower counts than the standard batches (B4, B5, B6). By T4, the standard batches exhibit higher counts compared to the experimental group. This plot effectively showcases the growth trends of microbial counts in Wiener sausages, allowing readers to quickly grasp the differences and changes during the storage period.

The scatter plot captures the TVC in log CFU/g for two time points (T0, storage day 0; T4, storage day 15) for all batches of Wiener sausages. The X-axis includes the B1, B2, and B3 experimental batches, as well as B4, B5, and B6 standard batches. The Y-axis contains TVC in log CFU/g, with points marked in light blue for T0 and light green for T4.

Statistical analysis through ANOVA revealed a significant TVC difference between T0 and T4 time points with a *p*-value less than 0.001. Batch variations did not produce statistically meaningful differences in TVC since the *p*-value was 0.333, which shows that batch effects do not explain much of the TVC variation.

Residuals analysis through the Shapiro–Wilk normality test yielded W = 0.80672 with a *p*-value of 0.01118. The ANOVA model’s residuals demonstrate significant deviation from normal distribution because the *p*-value (0.01118) falls below the threshold of 0.05 which leads to the rejection of the null hypothesis. The minimal value of the W statistic indicates significant non-normality within the residuals. Neither the log transformation nor the square root transformation succeeded in normalizing the residuals.

To determine significant differences between the medians of our groups, the Kruskal–Wallis test was employed (chi-squared = 8.3662, df = 1, *p*-value = 0.003823). Since the *p*-value (0.003823) is less than the conventional alpha level of 0.05, we rejected the null hypothesis, indicating significant differences in the medians of TVC between T0 and T4. Our analysis suggests that microbial counts significantly differ between Day 0 (T0) and Day 15 (T4). Results from Dunn’s test (chi-squared = 8.366197, Z = −2.892438, *p* = 0.001911324, adjusted *p* = 0.001911324) confirmed that the medians of total viable counts at T4 are significantly higher than at T0, supporting our earlier analyses indicating microbial growth over time.

We applied the Mann–Whitney U test (W = 2.5, *p*-value = 0.5066) to compare the TVC between the experimental and standard groups. While taking into consideration any problems with data distribution, this test shed light on the variations in TVC between the experimental and standard groups.

#### 3.1.2. Evaluation of pH and a_w_

The evolution of pH and a_w_ values on experimental batches (B1, B2, and B3) and standard batches (B4, B5, and B6) was recorded at day 0 of storage (T0) and the 15th day of storage (T4) (Table 3). The pH values ranged between 5.66 ± 0.40 and 5.90 ± 0.35 for experimental batches and from 5.89 ± 0.40 to 6.27 ± 0.35 for the standard batches, while the aw values for the experimental batches varied from 0.940 ± 0.002 to 0.952 ± 0.001 and for the standard batches from 0.942 ± 0.001 to 0.950 ± 0.001. The results were expressed as mean ± standard deviation (SD).

**Figure 4:**
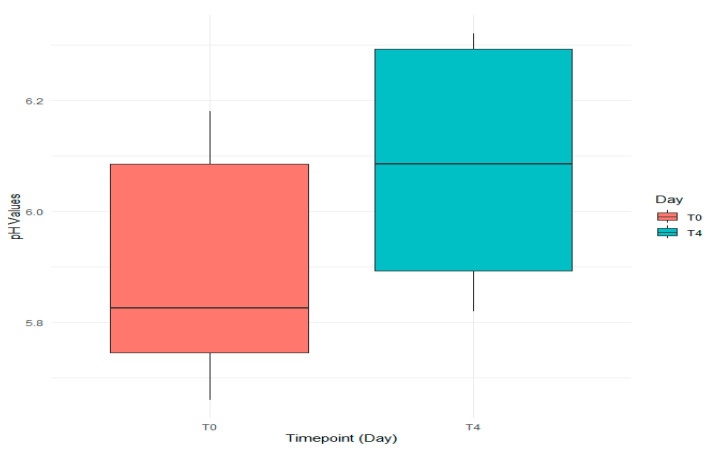
The pH levels of the Wiener sausages at T0 and T4.

A visual representation of pH levels (Figure 4) indicated that the median pH at T0 is lower than at T4, suggesting slight increases in pH values, potentially due to changes in storage conditions or microbial activity. The interquartile range (IQR) at both time points shows similar heights, indicating a consistent spread of data without outliers, reinforcing the reliability of the statistical analysis.

Measurements were taken across six different batches, labeled B1 to B6. The horizontal line inside each box shows the median pH value, the point where half of the values are higher and half are lower. The extent of each box represents the interquartile range (IQR), which is the range in which the middle 50% of the data fall. In the boxplot, the lines extending from the boxes, called whiskers, represent the range of pH values that are not considered unusual. Interestingly, there were no significantly unusual values, known as outliers, at either time point.

**Figure 5:**
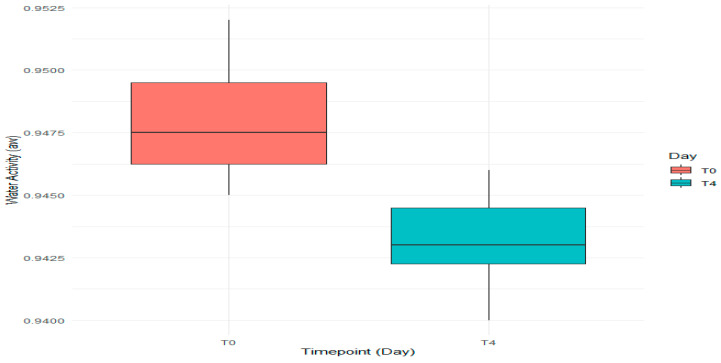
The water activity (a_w_) levels of Wiener sausages measured at two different times: on day 0 (T0) and again on day 15 (T4) of storage.

Similarly, the analysis of water activity levels (Figure 5) showed a decrease in average moisture content after 15 days of storage, with median aw dropping from approximately 0.9475 at T0 to 0.9430 at T4. The wider IQR for T0 suggests greater variability in moisture among initial batches compared to T4, where moisture loss appears more consistent.

In the plot, the boxes represent the range of values where the middle 50% of the data falls, while the horizontal line inside each box shows the median water activity. The data reveals that, on average, the water activity went down T0 to T4. The lines extending from the boxes, called whiskers, indicate the smallest and largest aw values that are still within the normal range, excluding any outliers.

The pH and aw data did not have any outliers based on the IQR method, which suggests that the measurements taken were accurate. For pH, Levene’s test indicated that the variances were homogeneous (F = 0.4367, *p* = 0.5237), and this was also confirmed for aw (F = 0.3571, *p* = 0.5634). Thus, we could safely apply ANOVA. A one-way ANOVA for pH showed no differences (F = 2.003, *p* = 0.187), which meant that T0 and T4 were similar. This required no subsequent analysis. On the other hand, the analysis of variance performed for water activity did show differences (F = 12.33, *p* = 0.00562), which meant that there were differences in moisture content for T0 and T4. This was supported by Duncan’s multiple range test which showed the water activity decreased from 0.9480 to 0.9432.

**Figure 6:**
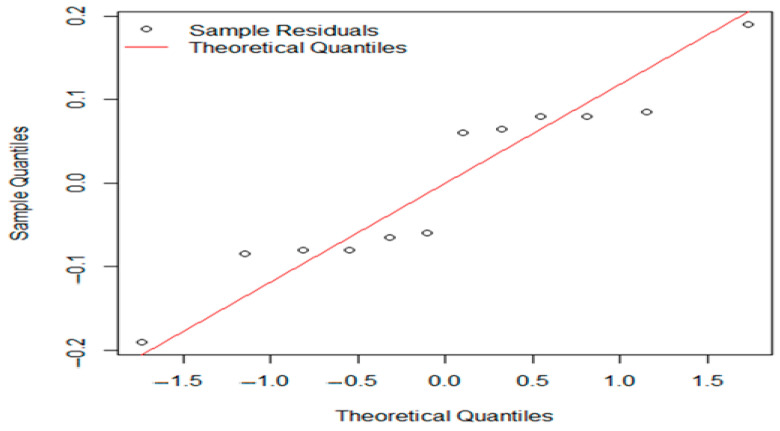
The residuals from Welch’s one-way ANOVA for pH levels.

**Figure 7:**
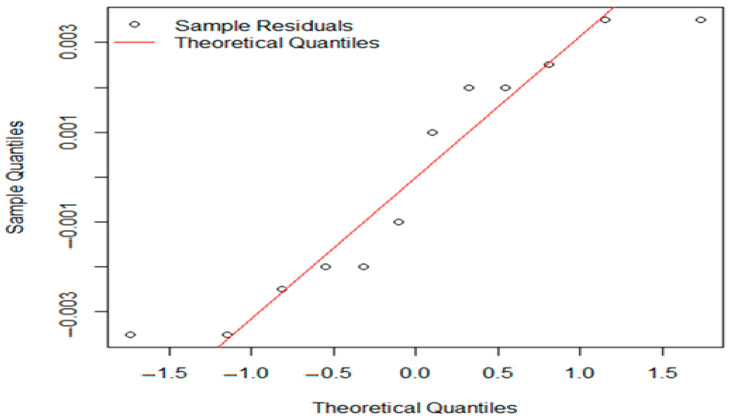
The residuals from Welch’s one-way ANOVA for aw values.

Conducting a further one-way ANOVA for the experimental and standard groups for both pH and aw, Levene’s test suggested unequal variance (*p* < 2.2 × 10^−16^). In this case, we applied Welch’s ANOVA followed by Games–Howell post hoc evaluation of the results. Welch’s ANOVA results showed no significant differences in pH (F = 4.7542, *p* = 0.1264) or aw (F = 0.47009, *p* = 0.7846) among batches at the 0.05 significance level, making further post hoc tests unnecessary. Q-Q plots of residuals from Welch’s ANOVA indicated that the residuals are approximately normally distributed (Figure 6 and Figure 7).

The plot checks if the residuals are normally distributed, which is an important assumption for the analysis. Open circles represent the residuals from the samples, positioned against their expected theoretical quantiles. If the points closely follow the red line, then the residuals are roughly normally distributed.

Open circles represent the residuals from the samples, positioned against their expected theoretical quantiles. If the points closely follow red line, then the residuals are roughly normally distributed.

### 3.2. Evaluation of L. monocytogenes Counts on Blank Samples of Wiener Sausages

The results obtained from blank samples of Wiener sausages for *L. monocytogenes* detection at T0 and T4 based on the VIDAS^®^ Listeria monocytogenes II test (LMO2 VIDAS^®^, bioMérieux, France) were compliant (negative), i.e., *L. monocytogenes* absent/25 g product (Table 4).

### 3.3. Results of the Behavior of L. monocytogenes in Artificially Inoculated Samples

The enumeration of *L. monocytogenes* was performed on inoculated experimental batches B1, B2, and B3 at the five defined times (Table 5). The standard deviation (SD) calculated at T0 was less than 0.3 log_10_ CFU/g per batch. This indicates that the inoculation of *L. monocytogenes* was homogeneous and that the test results can be used to calculate the growth potential/batch (Δ/batch) and to assess the growth potential of product F1 (marked as Δ/F1). The results of the *L. monocytogenes* count are expressed in log_10_ CFU/g. At T0, the means of pathogen counts varied between 2.66 ± 0.02 log_10_ CFU/g and 2.71 ± 0.05 log_10_ CFU/g. An increase was observed up to T3, where *L. monocytogenes* counts varied between 3.37 log_10_ CFU/g and 3.52 log_10_ CFU/g, followed by a slight decrease at T4, where its counts varied between 3.14 log_10_ CFU/g and 3.35 log_10_ CFU/g. The calculation of Δ/batch showed values between 0.71 log_10_ CFU/g and 0.82 log_10_ CFU/g. The growth potential of the experimental product F1 (Δ/F1) was 0.82 log_10_ CFU/g, which is above the limit of 0.5 log_10_ CFU/g [31]. F1 is classified as an RTE food able to support the growth of *L. monocytogenes* (category 1.2 of RTE food).

**Figure 8:**
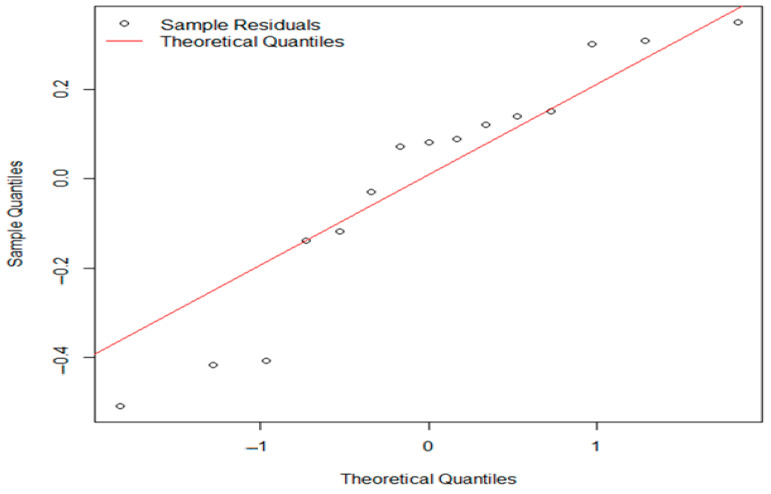
Q-Q plot of residuals from one-way ANOVA for *L. monocytogenes* (*Lm*) concentrations across experimental batches (B1, B2, B3).

Statistical analyses of *L. monocytogenes* concentrations in inoculated experimental batches stored under refrigerated conditions at 7 °C were performed using one-way ANOVA (F = 0.298, *p* = 0.748). The *p*-value far exceeds the common significance level of 0.05, indicating no statistically significant difference in *L. monocytogenes* concentrations among the different batches (B1, B2, B3). Consequently, we fail to reject the null hypothesis that all batch means are equal. The Shapiro–Wilk normality test (W = 0.90981, *p* = 0.1345) suggests that the data are normally distributed, and the residuals are also normally distributed (*p* > 0.05). Although the Q-Q plot shows some departure from the diagonal line, especially at the ends (as seen in Figure 8), the Shapiro–Wilk test indicates that the residuals still follow a normal distribution. Since our ANOVA results did not show any significant differences, these minor deviations in normality are unlikely to have a meaningful effect on our conclusions.

Open circles represent the residuals from the samples, positioned against their expected theoretical quantiles. If the points closely follow red line, then the residuals are roughly normally distributed.

The points show deviations from the diagonal, particularly at the tails, which may indicate deviations from normality. While the Shapiro–Wilk test suggests normality (*p* = 0.1345), the Q-Q plot reveals some departure from a normal distribution. Given that our ANOVA results are not significant, the assumption of normality in the residuals is unlikely to significantly affect our findings.

Levene’s test produced a very low F value of 0.0032 and a *p*-value of 0.9968, suggesting that the variances between the different groups are very similar to those within each group. This implies that the variances across the different batches (B1, B2, B3) are equal, indicating homoscedasticity. Additionally, Duncan’s test revealed no significant differences in *L. monocytogenes* concentrations among the batches, as they were all grouped together. Overall, both the ANOVA and the post hoc tests show that there are no significant differences among the groups.

Given the Q-Q plot’s deviations from normal distribution, we subsequently employed a non-parametric test for better validity. A Kruskal–Wallis test revealed no significant differences in *L. monocytogenes* concentrations among the batches, H(2) = 0.98, *p* = 0.6126. Dunn’s test for post hoc pairwise comparisons showed no statistically significant differences: (1) B1 vs. B2 yielded a Z = 0.495 with an unadjusted *p* = 0.6206 and a Bonferroni-adjusted *p* = 1.0000; (2) B1 vs. B3 produced a Z = −0.495 with an unadjusted *p* = 0.6206 and a Bonferroni-adjusted *p* = 1.0000; (3) B2 vs. B3 resulted in a Z = −0.990 with an unadjusted *p* = 0.3222 and a Bonferroni-adjusted *p* = 0.9666. These results show that there are no significant differences in *L. monocytogenes* concentrations between batches B1, B2, and B3, indicating that the levels of *L. monocytogenes* are quite similar across all the batches under the conditions we tested.

The Kruskal–Wallis test of *L. monocytogenes* at T0, T1, T2, T3, and T4 indicated no statistically significant differences (*p* = 0.2579). Specifically, mean log_10_ concentrations of *L. monocytogenes* at T0 were 2.71 ± 0.05 for B1, 2.66 ± 0.02 for B2, and 2.70 ± 0.10 for B3. As the experiment progressed to T1, T2, T3, and T4, concentrations increased, peaking at T3, where B3 recorded the highest concentration of 3.52 log_10_ CFU/g. These fluctuations did not reach statistical significance. *L. monocytogenes* concentrations remained stable across the measured time points.

**Figure 9:**
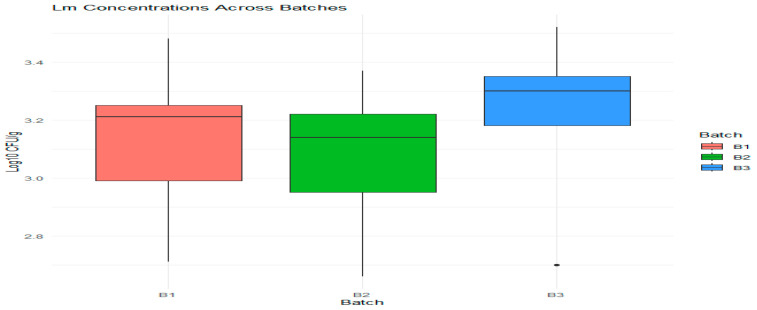
The concentrations of *L. monocytogenes* (*Lm*) measured in three experimental batches: B1, B2, and B3.

The visualization of *L. monocytogenes* concentrations across experimental batches (B1, B2, B3) shows overlapping interquartile ranges (Figure 9), and the absence of significant differences (as indicated by Duncan’s test) suggests that while there are differences in medians, they are not statistically significant. These results provide an awareness of the behavior of *Lm* in the environment we examined and suggest that other variables may contribute to its stability or increase over time.

The line inside each box represents the median value. Batch B3 showed the highest median concentration, while batch B2 had the lowest. The boxes illustrate the interquartile range, which includes the middle 50% of the data, and their height indicates how much variability there is in *L. monocytogenes* concentrations within each batch. Any dots that fall outside the “whiskers” are considered potential outliers, and for batch B1, there is one outlier that falls below the lower whisker.

The enumeration of *L. monocytogenes* was performed on the inoculated standard batches B4, B5, and B6 at the five defined times (Table 6). The standard deviation (SD) calculated at T0 was less than 0.3 log_10_ CFU/g per each sample. This indicates that the inoculation of *L. monocytogenes* was homogeneous and that the results of the test can be used to calculate the growth potential/batch (Δ/batch) and to assess the growth potential of product F2 (Δ/F2). The results of the *L. monocytogenes* count are expressed in log_10_ CFU/g. At T0, the pathogen counts varied between 2.78 ± 0.07 log_10_ CFU/g and 2.91 ± 0.02 log_10_ CFU/g. A continuous increase in the *L. monocytogenes* count was observed up to T4, where it ranged from 5.72 log_10_ CFU/g to 5.81 log_10_ CFU/g. The calculation of Δ/batch showed values between 2.90 and 2.94. The growth potential of the standard product F2 (Δ/F2) was 2.94 log_10_ CFU/g, thus exceeding the limit of 0.5 log_10_ CFU/g. F2 is classified as an RTE food able to support the growth of *L. monocytogenes* (category 1.2 of the RTE food).

**Figure 10:**
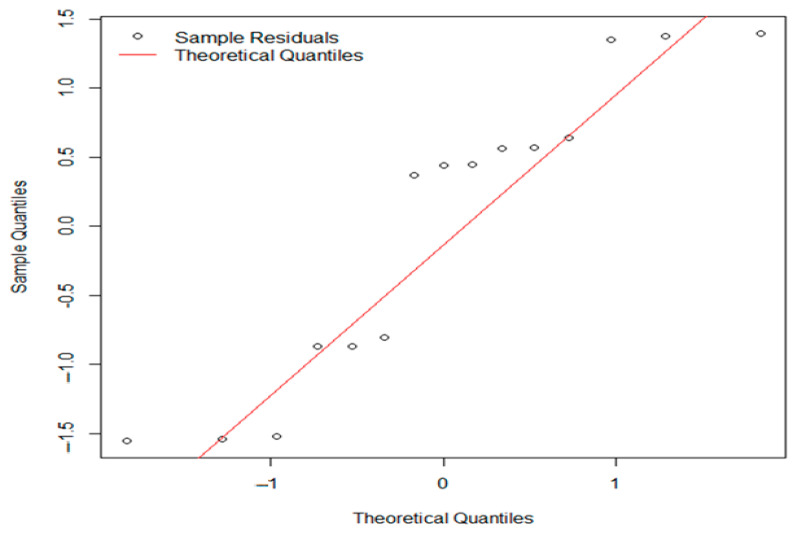
Analysis of residuals from one-way ANOVA for *L. monocytogenes* (*Lm*) concentrations across standard batches (B4, B5, and B6) by using Q-Q plot.

Statistical analyses of *L. monocytogenes* concentrations in inoculated standard batches stored under refrigerated conditions (7 °C) were conducted using one-way ANOVA (F = 0.012, *p* = 0.988). The *p*-value is significantly greater than the common significance level of 0.05, indicating no statistically significant difference in *L. monocytogenes* concentrations among the standard batches (B4, B5, and B6). We are unable to reject the null hypothesis, which states that the means for each batch are the same. The Shapiro–Wilk normality test showed W = 0.87553 with a *p*-value of 0.04071, indicating that the residuals might not be normally distributed, which suggests a potential issue with meeting the normality assumption (*p* < 0.05). This deviation from normality is further illustrated in the Q-Q plot, where several points fall away from the diagonal line (Figure 10). Even though our ANOVA results did not show significance, it seems that the normality assumption for the residuals is unlikely to have a substantial impact on our overall findings.

Open circles represent the residuals from the samples, positioned against their expected theoretical quantiles. If the points closely follow red line, then the residuals are roughly normally distributed.

Most of the data points closely follow the red line, suggesting that the sample data are approximately normally distributed.

Levene’s test (F = 0.0023, *p* = 0.9977) suggests that the variation within each group is nearly identical to the variance between the groups. This supports the assumption of homoscedasticity, indicating that the variances among the different standard batches (B4, B5, and B6) are equal. Additionally, Duncan’s test confirms that there are no significant differences in *L. monocytogenes* concentrations among B4, B5, and B6, as all batches were grouped together. Both ANOVA and post hoc tests indicate no significant differences among the batches.

We chose to take a closer look at the data using a non-parametric approach, specifically the Kruskal–Wallis test, since the Shapiro–Wilk test and the Q-Q plot indicated that the data did not follow a normal distribution. Our analysis showed that the *L. monocytogenes* concentrations in batches B4, B5, and B6 were not statistically different from each other (*p* = 0.8106), suggesting that the average concentrations in each batch are nearly the same. Furthermore, Dunn’s test for post hoc pairwise comparisons revealed no significant differences among the batches, reinforcing that the *L. monocytogenes* concentrations in B4, B5, and B6 are quite similar.

We then examined how *L. monocytogenes* concentrations changed over time by using a Kruskal–Wallis rank sum test for the different time points (T0, T1, T2, T3, and T4). The results showed significant changes in *L. monocytogenes* concentrations, indicated by a chi-squared statistic of 13.5 with four degrees of freedom and a *p*-value of 0.009074.

Dunn’s test for post hoc pairwise comparisons then showed that there was only one significant difference between T0 and T4, with an adjusted *p*-value of 0.01015 and a Z-value of −3.286. This suggests that at T0, *L. monocytogenes* concentrations were substantially lower than at T4. The comparisons that did not reveal significant differences included T0 vs. T1 (Z = −0.822, adjusted *p* = 1.000), T0 vs. T2 (Z = −1.643, adjusted *p* = 1.000), T1 vs. T2 (Z = −0.822, adjusted *p* = 1.000), T1 vs. T3 (Z = −1.643, adjusted *p* = 1.000), and T2 vs. T3 (Z = −0.822, adjusted *p* = 1.000). This indicates that, even without significant changes detected between these time points, the leap between T0 and T4 remains quite significant. While there were no notable changes in the other time periods, the overall analysis clearly shows that *L. monocytogenes* concentrations tend to increase over time, particularly from T0 to T4. This really highlights how important storage time is for the behavior of *L. monocytogenes*. It also points out the need for further exploration into the various factors that influence its growth dynamics. Understanding these factors could help us better manage and control *L. monocytogenes* levels in different environments, which is essential for ensuring food safety and quality.

**Figure 11:**
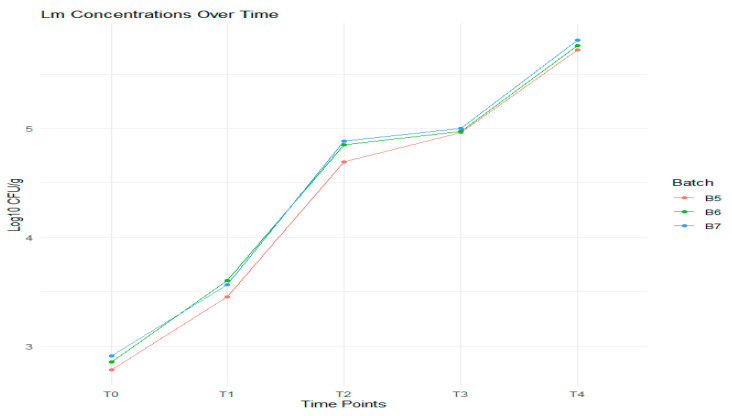
Dynamics of *L. monocytogenes* (*Lm*) in experimental batches B5, B6, and B7 measured at five time points.

The *L. monocytogenes* dynamics in the experimental batches is presented in Figure 11.

Observations indicate that *L. monocytogenes* vary significantly over time, reflecting microbial growth kinetics and potential environmental influences.

In the experimental batches, the mean concentrations of *L. monocytogenes* increase gradually from T0 to T3, followed by a slight decrease at T4. The SDs indicate that *L. monocytogenes* is relatively stable, with low variability across time points, and the variance values support this stability, being close to zero, particularly at T0 and T2 (Table 7). In contrast, the means for the standard batches are significantly higher across all time points compared to the experimental batches, suggesting differences in growth or treatment conditions.

Although the standard deviations in the standard batches remain low, they exhibit slightly higher variability compared to the experimental batches, and the variance reflects this mild deviation from the means (Table 7). To assess whether the observed differences between experimental and standard batches are statistically significant, we plan to conduct further statistical tests.

**Figure 12:**
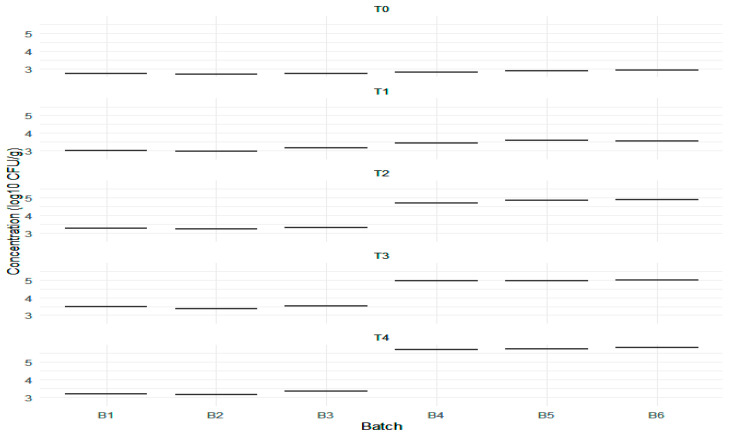
The concentrations of *L. monocytogenes* in log_10_ CFU/g across six batches (B1, B2, B3 for experimental and B4, B5, B6 for standard) at five time points (T0 to T4).

To evaluate the differences in *L. monocytogenes* concentrations between experimental batches (B1, B2, and B3) and standard batches (B4, B5, and B6) at each time point (T0, T1, T2, T3, and T4), we employed the Kruskal–Wallis test, a non-parametric method for assessing differences among two or more independent groups, and the Mann–Whitney U test to compare two independent groups (experimental vs. standard batches) for each specific time point. The Kruskal–Wallis test resulted in a chi-squared statistic of 10.006 with five degrees of freedom (df), yielding an associated *p*-value of 0.07505, which indicates no statistically significant differences in the concentrations among the groups (*p* > 0.05). The Mann–Whitney U test similarly revealed no significant differences in *L. monocytogenes* concentrations between the experimental and standard batches at any time points, with *p*-values for T0, T1, T2, T3, and T4 all equaling 0.1. All these *p*-values exceeded the significance level of 0.05, suggesting that the median concentrations of *L. monocytogenes* among the groups were comparable across the evaluated moments. Observations from the boxplots indicated that concentrations in the standard batches (B4, B5, and B6) were generally higher than those in the experimental batches (B1, B2, and B3) at all time points, reflecting a consistent trend (Figure 12). However, the overlapping interquartile ranges indicate considerable overlap in *L. monocytogenes* concentrations between groups, corroborating the Mann–Whitney U test findings of no significant differences.

Each row represents a specific day of storage, highlighting how concentration levels vary over time. The line within each box indicates the median concentration, while the edges represent the interquartile range, illustrating the distribution of data. Overall, concentrations tend to rise during storage, revealing distinct growth patterns between experimental and standard batches.

Interestingly, while the standard batches showed higher mean concentrations, the lack of statistical significance underscores the importance of considering variability and sample size when interpreting the results. The visual representations provided by the boxplots enhance our understanding of data distribution and highlight potential trends that may warrant further investigation.

## 4. Discussion

As RTE meat products, Wiener sausages must comply with the provisions of Regulation (EC) No. 2073/2005 regarding *L. monocytogenes*. The growth potential (Δ) was used to study the behavior of this pathogen throughout the shelf life of the product, under conditions of various preservatives and antioxidants. The *L. monocytogenes* growth in food could be influenced by various factors, as intrinsic as pH, a_w_, and aerobic mesophilic microflora, and as extrinsic as the temperature of storage, the gas composition of modified atmosphere in package, and the level of contamination.

The spoilage microflora level reflects the effects of time and temperature of storage for batches of Wiener sausages. The aerobic mesophilic microflora on control samples, expressed as TVC (total viable count) in log CFU/g, showed an increase during the 15 days of storage for all batches. There is no significant difference in TVC between the experimental group (B1, B2, and B3) and the standard group (B4, B5, and B6) based on the Mann–Whitney U test results. Although the mean TVC values indicate that the standard group has a higher average, this difference is not statistically significant. The calculated effect size (r = 0.0067) suggests a small effect, implying that while the difference in microbial counts was not statistically significant, it may warrant further exploration, especially in contexts where microbial loads are critical.

At T0, the levels of TVC of B1, B2, and B3 were similar to the levels of TVC of B4, B5, and B6 under refrigerated storage conditions; the highest value (2.53 ± 0.35 log CFU/g) was recorded for batch B5. At T4, the standard batches B4, B5, and B6 had higher values under refrigerated storage conditions; the maximum value (3.85 log CFU/g) was recorded on batch B5, as an effect of storage time on spoilage microorganism growth. The standard batches B4, B5, and B6 showed a growth of aerobic mesophilic flora during the 15 days of storage. On the other hand, the TVC values recorded for batches B1, B2, and B3 over 15 days of storage under refrigerated conditions, reflecting the effect of the combination of PFJ and HBE used in the experimental products, showed a smaller increase. The antimicrobial action of PFJ and HBE is significant, ensuring microbiological stability and preventing product spoilage throughout the shelf life of the product. Similar results were reported by various studies regarding the effect of natural preservatives and polyphenols [28,33,48]. Use of hawthorn berry extract in combination with fermented parsley juice induced a reduction in spoilage microorganism growth in experimental batches, as a result of several constituent compounds.

The results of the physicochemical parameters of the control samples (Table 3) indicated that a pH value > 5 in all batches over 15 days of refrigeration temperature is a favorable feature that allows *L. monocytogenes* multiplication. The pH values showed an increase during the 15 days of shelf life for all batches of Wiener sausages. The increase in pH of meat and meat products has been reported in various studies as a result of the production of volatile compounds (i.e., ammonia) and microbial catabolites during shelf life under refrigerated conditions [33,49,50,51,52]. The pH values in experimental batches B1, B2, and B3 are higher after 15 days of storage. A slight increase in pH was observed in the experimental batches compared with the standard batches B4, B5, and B6, due to the inhibitory effect of the antimicrobial compounds in HBE and PFJ on the growth and multiplication of spoilage microorganisms that metabolize basic nitrogen compounds [33].

Based on statistical data, it was observed that the median pH level increased from the initial measurement (T0) to the last one (T4), suggesting that the Wiener sausages may become a bit more acidic as they are stored over time (Figure 4). The descriptive statistics reveal that experimental batches are more acidic compared to the standard batches, which exhibit higher variability in pH.

In our study, a_w_ was ≥0.94 in all batches, which has no effect on the potential growth of *L. monocytogenes* during 15 days of shelf life under refrigeration conditions at 7 °C. Several studies demonstrated that growth inhibition could be possible in conditions of pH ≤ 5 and a_w_ ≤ 0.94 of the product, under refrigeration conditions [53]. The statistical analysis showed a decrease of a_w_ that could mean that the Wiener sausages lost some moisture or that their structure changed during the storage time (Figure 5).

*L. monocytogenes* detection indicates that all blank samples are not contaminated, with the pathogen being absent/25g at T0 and T4 (Table 4). These results represent a marker which allows to perform evaluation of *L. monocytogenes* growth potential. In the case of natural contamination of the samples, the level of contamination shall be less than or equal to the level of artificial contamination in order to assess the behavior of *L. monocytogenes* during the shelf life of the product [31,54]. The potential contamination of RTE food can occur frequently, with post-heat recontamination being common. The contribution of the manufacturing environment as a source of *L. monocytogenes* contamination has been demonstrated previously, as the microbe is widespread and survives under different conditions of temperature and pH [55].

The study of *L. monocytogenes* behavior in experimental product F1, based on a challenge test, showed an increase in *L. monocytogenes* in log_10_ CFU/g during the first 10 days of the assay (T0–T3), followed by a slight reduction at the end of the assay (T4), and the calculated growth potential value Δ/F1 (0.82 log_10_ CFU/g) indicates that *L. monocytogenes* growth is still possible. With the addition of 50 ppm phenolic-rich HBE and 50 ppm of PFJ, the pathogen showed slight growth inhibition in batches of experimental product F1.

Phenolic extracts from various fruits and vegetables have been used as preservatives for meat and meat products, particularly for their antimicrobial and antioxidant activity [33,48,49,56,57]. Several studies have evaluated and demonstrated the antimicrobial mechanisms by inducing important changes in cell membrane permeability, loss of bacterial cell constituents, and bacterial cell death [58,59]; pointed to the role of phenolic compounds in preventing biofilm formation, a challenging issue in relation to *L. monocytogenes* [60]; or demonstrated the effect of various phenolic extracts, e.g., the use of 2% cranberry pomace as a preservative in cooked ham induced a significant growth-slowing of this foodborne pathogen [61]. The antimicrobial effect of phenolic compounds has mostly been to reduce or inhibit microorganisms in food, including foodborne pathogens [13,15,20,62]. However, the activity of phenolic compounds against *L. monocytogenes* depends on their dose, structure, and chemical composition, but it is also influenced by the food matrix, i.e., the fat and protein composition [59].

The PFJ has been used in experimental batches as a natural source of nitrite. The efficacy of the use of nitrite as a preservative in the meat industry has been demonstrated by various studies from many years ago, including its antimicrobial effect [63]. The bactericidal effect of nitrite from natural sources against foodborne pathogens, i.e., *Clostridium botulinum*, *Escherichia coli*, and *L. monocytogenes*, depends on the food matrix, including the ingredients, pH, sodium chloride content, and heat treatment [62,64,65]. A significant reduction in *L. monocytogenes* growth during storage was observed in studies regarding the effect of parsley extract powder in mortadella-type sausages, with the effect being in direct relation to the quantity of powder [62,66].

The study of *L. monocytogenes* behavior in standard product F2, based on a challenge test, showed an increase over 15 days of testing (T0–T4). The calculated growth potential value Δ/F2 (2.94 log_10_ CFU/g) showed that *L. monocytogenes* growth is still available under conditions of added 50 ppm sodium nitrite and 50 ppm of sodium ascorbate. The study of Hospital et al. (2017) on the antimicrobial action of sodium nitrite and sodium ascorbate showed that the mixture had no effect on the growth potential of *L. monocytogenes* in meat products [67]. Some research regarding the nitrite effect against *L. monocytogenes* showed that growth pathogens appear to have been reduced in fermented dry sausages as a result of pH, sodium chloride, and sodium nitrite combined action and a low a_w_ [64]. Another study highlighted the antimicrobial effect of nitrites combined with sodium diacetate and sodium lactate in meat products, with a concerted action against *L. monocytogenes* [68,69].

Both challenged products, F1 and F2, demonstrated the ability to support *L. monocytogenes* growth over 15 days of testing, with a growth potential above the threshold value of 0.5 log_10_ CFU/g. Considerably higher potential growth was reported for the standard product F2, in comparison with experimental product F1, where the value was lower.

The addition of PFJ and HBE as natural preservatives in the experimental product was associated with a decrease in *L. monocytogenes* without eliminating the potential growth during shelf life. According to Regulation (EC) No. 2073/2005, based on the growth potential values Δ/F1 and Δ/F2, Wiener sausages are an RTE food capable of supporting *L. monocytogenes* growth. The pathogen must not be detected in 25 g of the sample prior to the food leaving the direct control of the food business operator who produced it, according to legal provisions for this RTE food category [8].

The difference between the two product types on the growth potential of *L. monocytogenes* underlines the antimicrobial activity developed by the compounds of fermented parsley root juice and hawthorn berry phenolics in Wiener sausages, as RTE meat product, during the shelf life stored at 7 °C. The use of fermented parsley root juice and hawthorn berry phenolics as natural preservatives in meat processing technology may be a useful alternative to synthetic additives, based on the results of our experiment.

The combined results of the Mann–Whitney U test and the visual data representation in boxplots indicate that, although there are observable differences in *L. monocytogenes* concentrations between experimental and standard batches, these differences are not statistically significant. Future studies could benefit from larger sample sizes or the inclusion of additional factors to better elucidate influences on *L. monocytogenes* concentrations in different batch preparations.

## 5. Conclusions

*L. monocytogenes*, as a foodborne pathogen, represents a major microbiological hazard, and the assessment of its growth potential through the shelf life of RTE food is mandatory for the guarantee of food safety, and, implicitly, the health of consumers. The composition of various vegetables and fruits studied showed that they could be valuable alternative sources for sodium nitrite and sodium ascorbate used as food additives in the meat industry. The nitrite and phenolic compounds of these natural ingredients showed antimicrobial activity against foodborne pathogens, including *L. monocytogenes*. The behavior of *L. monocytogenes* in Wiener sausages treated with fermented parsley root juice and hawthorn berry phenolics underlines that natural ingredients could act as preservatives that ensure microbiological safety during the shelf life of the product. However, it is necessary to adjust their dose and combinations, such that the RTE products are unable to support *L. monocytogenes* growth during their shelf life.

## Figures and Tables

**Table 1:** The types of Wiener sausage product formulations used in the study. Fermented parsley juice—PFJ; hawthorn berry extract—HBE; sodium nitrite—N; sodium ascorbate—A; F1—experimental product; F2—standard product; B1–B3—the code of experimental batches of Wiener sausages; B4–B6—the code of standard batches of Wiener sausages.

Product	Batch	Preservatives and Antioxidants			
		PFJ (ppm NO_2_^−^)	HBE (ppm GAE)	N (ppm NO_2_^−^)	A (ppm Ascorbate)
F1	B1	50	50		
	B2	50	50		
	B3	50	50		
F2	B4			50	50
	B5			50	50
	B6			50	50

**Table 2:** Aerobic mesophilic microflora counts and descriptive statistics in control samples of Wiener sausages. B1–B3—code of experimental batches of Wiener sausages; B4–B6—code of standard batches of Wiener sausages; TVC—total viable count; T0—day 0 of storage; T4—15th day of storage.

Batch	TVC at T0 (log CFU/g)			TVC at T4 (log CFU/g)		
	Mean	SD	Variance (log CFU/g)^2^	Mean	SD	Variance (log CFU/g)^2^
B1	2.35	0.40		3.30	0.30	
B2	2.43	0.35		3.23	0.35	
B3	2.50	0.40		3.30	0.35	
**Experimental Group** **(B1, B2, B3)**	**2.43**	**0.0751**	**0.00563**	**3.28**	**0.0404**	**0.00163**
B4	2.50	0.40		3.75	0.35	
B5	2.53	0.35		3.85	0.35	
B6	2.37	0.45		3.70	0.30	
**Standard Group** **(B4, B5, B6)**	**2.47**	**0.0850**	**0.00723**	**3.77**	**0.0764**	**0.00583**

**Table 3:** pH and a_w_ values on control samples of Wiener sausages. B1–B3—code of experimental batches of Wiener sausages; B4–B6—code of standard batches of Wiener sausages; a_w_—water activity; T0—day 0 of storage; and T4—15th day of storage.

Batch	Day	pH			a_w_		
		Mean	SD	Variance	Mean	SD	Variance
B1	T0	5.76	0.45		0.952	0.001	
	T4	5.89	0.35		0.945	0.002	
B2	T0	5.66	0.40		0.947	0.001	
	T4	5.82	0.40		0.940	0.002	
B3	T0	5.74	0.35		0.948	0.002	
	T4	5.90	0.35		0.943	0.001	
**Experimental Group** **(B1, B2, B3)**		**5.80**	**0.0929**	**0.00863**	**0.946**	**0.00417**	**0.0000174**
B4	T0	6.15	0.30		0.950	0.001	
	T4	6.32	0.45		0.946	0.001	
B5	T0	6.18	0.35		0.945	0.003	
	T4	6.30	0.40		0.943	0.001	
B6	T0	5.89	0.40		0.946	0.0002	
	T4	6.27	0.35		0.942	0.001	
**Standard Group** **(B4, B5, B6)**		**6.18**	**0.159**	**0.0254**	**0.945**	**0.00280**	**0.00000787**

**Table 4:** *L. monocytogenes* counts on blank samples of Wiener sausages. B1–B3—code of experimental batches of Wiener sausages; B4–B6—code of standard batches of Wiener sausages; T0—day 0 of storage; and T4—15th day of storage.

Time (Day)	B1	B2	B3	B4	B5	B6
T0	absent/25 g	absent/25 g	absent/25 g	absent/25 g	absent/25 g	absent/25 g
	absent/25 g	absent/25 g	absent/25 g	absent/25 g	absent/25 g	absent/25 g
	absent/25 g	absent/25 g	absent/25 g	absent/25 g	absent/25 g	absent/25 g
T4	absent/25 g	absent/25 g	absent/25 g	absent/25 g	absent/25 g	absent/25 g
	absent/25 g	absent/25 g	absent/25 g	absent/25 g	absent/25 g	absent/25 g
	absent/25 g	absent/25 g	absent/25 g	absent/25 g	absent/25 g	absent/25 g

**Table 5:** Behavior of *L. monocytogenes* in inoculated experimental batches, during storage under refrigerated conditions at 7 °C. *Lm*—*L. monocytogenes*; B1–B3—code of experimental batches of Wiener sausages; T0—day 0 of storage; T1—3rd day of storage; T2—5th day of storage; T3—10th day of storage; T4—15th day of storage; Δ—growth potential; and F1—experimental product.

Batch	T0		T1	T2	T3	T4	Δ/Batch log_10_ CFU/g	Δ/F1 log_10_ CFU/g
	*Lm* (log_10_ CFU/g)	Mean ± SD	*Lm* (log_10_ CFU/g)	*Lm* (log_10_ CFU/g)	*Lm* (log_10_ CFU/g)	*Lm* (log_10_ CFU/g)		
B1	2.66	2.71 ± 0.05	2.99	3.25	3.48	3.21	3.48−2.71 = 0.77	0.82
	2.76							
	2.72							
B2	2.63	2.66 ± 0.02	2.95	3.22	3.37	3.14	3.37−2.66 = 0.71	
	2.67							
	2.68							
B3	2.59	2.70 ± 0.10	3.18	3.30	3.52	3.35	3.52−2.70 = 0.82	
	2.72							
	2.79							

**Table 6:** Behavior of *L. monocytogenes* in inoculated standard batches, during storage under refrigerated conditions at 7 °C. *Lm*—*L. monocytogenes*; B4–B6—code of standard batches of Wiener sausages; T0—day 0 of storage; T1—3rd day of storage; T2—5th day of storage; T3—10th day of storage; T4—15th day of storage; Δ—growth potential; and F2— standard product.

Batch	T0		T1	T2	T3	T4	Δ/Batch log_10_ CFU/g	Δ/F2 log_10_ CFU/g
	*Lm* (log_10_ CFU/g)	Mean ± SD	*Lm* (log_10_ CFU/g)	*Lm* (log_10_ CFU/g)	*Lm* (log_10_ CFU/g)	*Lm* (log_10_ CFU/g)		
B4	2.79	2.78 ± 0.07	3.45	4.69	4.96	5.72	5.72−2.78 = 2.94	2.94
	2.84							
	2.70							
B5	2.83	2.85 ± 0.02	3.60	4.85	4.97	5.76	5.76−2.85 = 2.91	
	2.87							
	2.86							
B6	2.88	2.91 ± 0.02	3.56	4.88	5.00	5.81	5.81−2.91 = 2.90	
	2.93							
	2.92							

**Table 7:** Mean, standard deviation, and variance of experimental batches (B1, B2, B3) and standard batches (B4, B5, B6) inoculated with *L. monocytogenes* and refrigerated at 7 °C.

Batch	T0	T1	T2	T3	T4
	*Lm* (log_10_ CFU/g) (mean ± SD)	*Lm* (log_10_ CFU/g)	*Lm* (log_10_ CFU/g)	*Lm* (log_10_ CFU/g)	*Lm* (log_10_ CFU/g)
**Experimental Batch**					
B1	2.71	2.99	3.25	3.48	3.21
B2	2.66	2.95	3.22	3.37	3.14
B3	2.70	3.18	3.30	3.52	3.35
***Mean***	***2.69***	***3.04***	***3.26***	***3.46***	***3.23***
***Standard Deviation***	***0.0265***	***0.123***	***0.0404***	***0.0777***	***0.107***
***Variance***	***0.000700***	***0.0151***	***0.00163***	***0.00603***	***0.0114***
**Standard Batch**					
B4	2.78	3.45	4.69	4.96	5.72
B5	2.85	3.60	4.85	4.97	5.76
B6	2.91	3.56	4.88	5.00	5.81
***Mean***	***2.85***	***3.54***	***4.81***	***4.98***	***5.76***
***Standard Deviation***	***0.0651***	***0.0777***	***0.102***	***0.0208***	***0.0451***
***Variance***	***0.00423***	***0.00603***	***0.0104***	***0.000433***	***0.00203***

## Data Availability

Upon reasonable request, the corresponding author will provide the datasets used and analyzed in this work.
